# Co-expression of miRNA players in advanced laryngeal carcinoma–insights into the roles of miR-93-5p, miR-145-5p, and miR-210-3p

**DOI:** 10.17305/bb.2024.10947

**Published:** 2024-09-23

**Authors:** Silva Kyurkchiyan, Veronika Petkova, Gergana Stancheva, Iglika Stancheva, Stoyan Dimitrov, Venera Dobriyanova, Diana Popova, Radka Kaneva, Todor M Popov

**Affiliations:** 1 Molecular Medicine Center, Department of Medical Chemistry and Biochemistry, Medical Faculty, Medical University of Sofia, Sofia, Bulgaria; 2 Department of Ear, Nose and Throat Diseases, University Multiprofile Hospital for Active Treatment “Tsaritsa Yoanna - ISUL,” Medical University of Sofia, Sofia, Bulgaria

**Keywords:** Laryngeal squamous cell carcinoma (LSCC), miRNA, miR-93-5p, miR-145-5p, miR-210-3p, co-expression, in silico, Gene Ontology annotation

## Abstract

Advanced laryngeal squamous cell carcinoma (LSCC) is the second most prevalent type of head and neck squamous cell carcinoma (HNSCC). Identifying microRNAs (miRNAs) related to key regulatory molecules or mechanisms could offer an alternative approach to developing new treatment strategies. The aim of our study is to evaluate significant correlations among deregulated miRNAs in advanced laryngeal carcinoma and to analyze, in silico, their strength of association, targets, and the most deregulated pathways. Several miRNAs demonstrated promising co-expression results, specifically miR-93-5p, miR-145-5p, and miR-210-3p. Their expressions were explored and further validated in a large set of *in vivo* advanced LSCC samples, which were subsequently used for bioinformatics and enrichment analyses. Our results highlight the significant roles of miR-93-5p, miR-145-5p, and miR-210-3p in regulating major pathways linked to the cell cycle via epithelial-to-mesenchymal transition (EMT), PI3K/Akt signaling, hypoxia, metabolism, apoptosis, angiogenesis, and metastasis. The associations between the expressions of these miRNAs and patients’ clinical features could be central to the progression of advanced LSCC. Overall, our study provides important insights into the co-expression and regulatory networks of miR-93-5p, miR-145-5p, and miR-210-3p in advanced laryngeal carcinoma, underscoring their potential as therapeutic targets or biomarkers for this aggressive cancer. Further research is needed to elucidate the specific mechanisms through which these miRNAs contribute to the pathogenesis and progression of laryngeal carcinoma.

## Introduction

Laryngeal cancer, a prevalent form of head and neck malignancy, ranks second globally within this category. It is predominantly represented by squamous cell carcinoma (SCC), which accounts for over 95% of cases. The primary risk factors for laryngeal carcinoma include tobacco use and alcohol consumption. While HPV infection was not historically considered a major risk factor, it has now been recognized as contributing to an increased risk of laryngeal cancer, alongside other factors such as occupational exposure [1]. Despite ongoing advancements in treatment, the prognosis for advanced laryngeal carcinoma remains challenging, with survival rates showing limited improvement over recent decades. Current treatment options—surgical interventions, radiotherapy, chemotherapy, and immunotherapy—can be used alone or in combination [2]. However, their effectiveness varies across populations, often influenced by economic disparities. A comprehensive review by Kordbacheh and Farah emphasized the role of molecular therapies in head and neck cancers [3], but the field of personalized medicine, particularly in oncology, still lacks validated biomarkers to guide treatment decisions. The FDA has approved molecular therapies, such as anti-EGFR and PD-1 inhibitors, for head and neck SCC (HNSCC). Key tests like p16 immunohistochemistry and PD-L1 expression have been associated with improved survival and prognosis [4], and they inform therapeutic strategies [5]. However, the lack of universally accepted clinical treatment protocols and definitive biomarkers for laryngeal cancer continues to hinder personalized patient care, leading to suboptimal outcomes and potential adverse effects. The discovery of microRNAs (miRNAs) more than two decades ago marked a pivotal shift in cancer research [6]. Their distinct expression patterns between healthy and diseased tissues, and their role in regulating complex biological processes (BPs) have sparked significant interest. To date, thousands of studies have explored the role of miRNAs in various diseases, including cancer. Several miRNAs have been identified as precise biomarkers, with some even progressing to clinical trials. However, the integration of miRNAs into routine clinical diagnostics remains limited. For example, miR-122 (Miravirsen), initially developed for hepatitis C treatment, remains in the research phase and has not yet been widely applied [7]. Due to its interactions with multiple oncogenes, miR-145 is considered a promising candidate for miRNA replacement therapy aimed at controlling metastasis. Recent studies have begun to associate miRNA expression with treatment outcomes in head and neck cancers [8]. For instance, miR-210 has been linked to chemotherapy resistance in laryngeal cancer [9], while miR-93 has been connected to poor prognosis through its targeting of cyclin G2 (CCNG2) [10]. Additionally, miR-31 has shown promise as a diagnostic marker for laryngeal SCC (LSCC) [11]. Moreover, miR-145 has been associated with cancer invasion and nodal metastasis, contributing to worse survival outcomes in OSCC [12]. As a result, miR-145 has emerged as a potential candidate for miRNA replacement therapy aimed at controlling metastasis [13]. A recent study based on The Cancer Genome Atlas (TCGA) data has explored co-expression networks in various cancers, including those affecting the brain, ovary, breast, and kidney [13]. Through pathway enrichment and gene ontology (GO) analyses, the study highlighted both commonalities and differences in co-expression networks among these cancer types, underscoring the distinct mechanisms of carcinogenesis across different tumors.

Our research focuses on enhancing the understanding of laryngeal carcinogenesis, specifically by identifying molecular biomarkers and key pathways involved in this process. The aim of our study is to investigate global miRNA profiling and evaluate correlations among the most deregulated miRNAs in advanced laryngeal carcinoma. We plan to use in silico methods to explore the strength of these associations, investigate miRNA co-expression networks, and analyze their shared and distinct target genes and pathways. This research may uncover disrupted regulatory and signaling pathways in laryngeal cancer, potentially leading to the development of more reliable therapeutic biomarkers and strategies. A deeper understanding of miRNA functions within these pathways could reveal vulnerabilities and lead to more personalized treatment approaches. Furthermore, we aim to investigate correlations between miRNA expressions and clinicopathological features.

## Materials and methods

### Patient sample collection

Fresh-frozen tumor and adjacent healthy tissues were collected from 131 advanced LSCC patients. These patients were hospitalized at the Ear, Nose, and Throat Department of University Hospital “Tsaritsa Yoanna” – ISUL, Sofia, Bulgaria, between 2015 and 2023. During surgery, within 1–3 min of resecting the tissue samples, they were frozen in liquid nitrogen (--196 ^∘^C). The samples were transported to the laryngeal tissue biobank of the Molecular Medicine Center, Sofia, Bulgaria, within 1–2 days and stored at --80 ^∘^C. None of the patients had undergone chemotherapy or radiotherapy prior to surgery. All patients signed written informed consent before participating in the study. Ethics approval was obtained from the Ethics Committee of the Medical University of Sofia (approval Nos. BK-297/14.04.2015, BK-373/11.04.2016, 7836/17.11.2020, and 4584/04.07.2023).

**Table 1:** miRNA names and their GeneGlobe IDs, catalog numbers (Qiagen, Germany) and the number of the transcripts that they amplificated

**Target name**	**GeneGlobe ID**	**Catalogue number**	**Target transcript amplification**
miR-93-5p	YP00204715	339306	MIMAT0000093
miR-145-5p	YP00204483	339306	MIMAT0000437
miR-210-3p	YP00204333	339306	MIMAT0000267
RNU6	SBM1036016	none	ENSMUST00000178023

miRNAs: MicroRNAs.

### Histopathological and clinical characteristics

In addition to fresh-frozen samples, tissue samples from the 131 LSCC patients, preserved as formalin-fixed paraffin-embedded (FFPE) specimens, were analyzed for histopathological characteristics. Tissues were preserved in 10% neutral buffered formalin for 24–48 h at room temperature, with sections cut to a thickness of 5–6 µm. These analyses followed the gold standard for solid tumor diagnosis and included assessments of tissue origin, cancer staging, grading (tumor aggressiveness), lymph node metastasis, and cell differentiation. The anatomical location of neoplasms was also documented during surgical procedures. Clinical information such as sex, age, tobacco and alcohol use, family history, and exposure to harmful environmental factors was collected alongside histopathological data.

### Extraction of total RNA

Total RNA (tRNA) was extracted from fresh-frozen tumor and adjacent healthy tissues using the miRNeasy Micro Kit (Qiagen, Germany) following the manufacturer’s protocol. The quantity of tRNA was assessed using a NanoDrop 2000 (ThermoFisher Scientific, USA) and a Qubit v2.0 fluorometer (ThermoFisher Scientific, USA). RNA integrity was evaluated using an RNA integrity number (RIN) measured on an Agilent 2100 Bioanalyzer (Agilent Technologies, USA). All samples included in the study had RIN values greater than 5.

**Figure 1.**
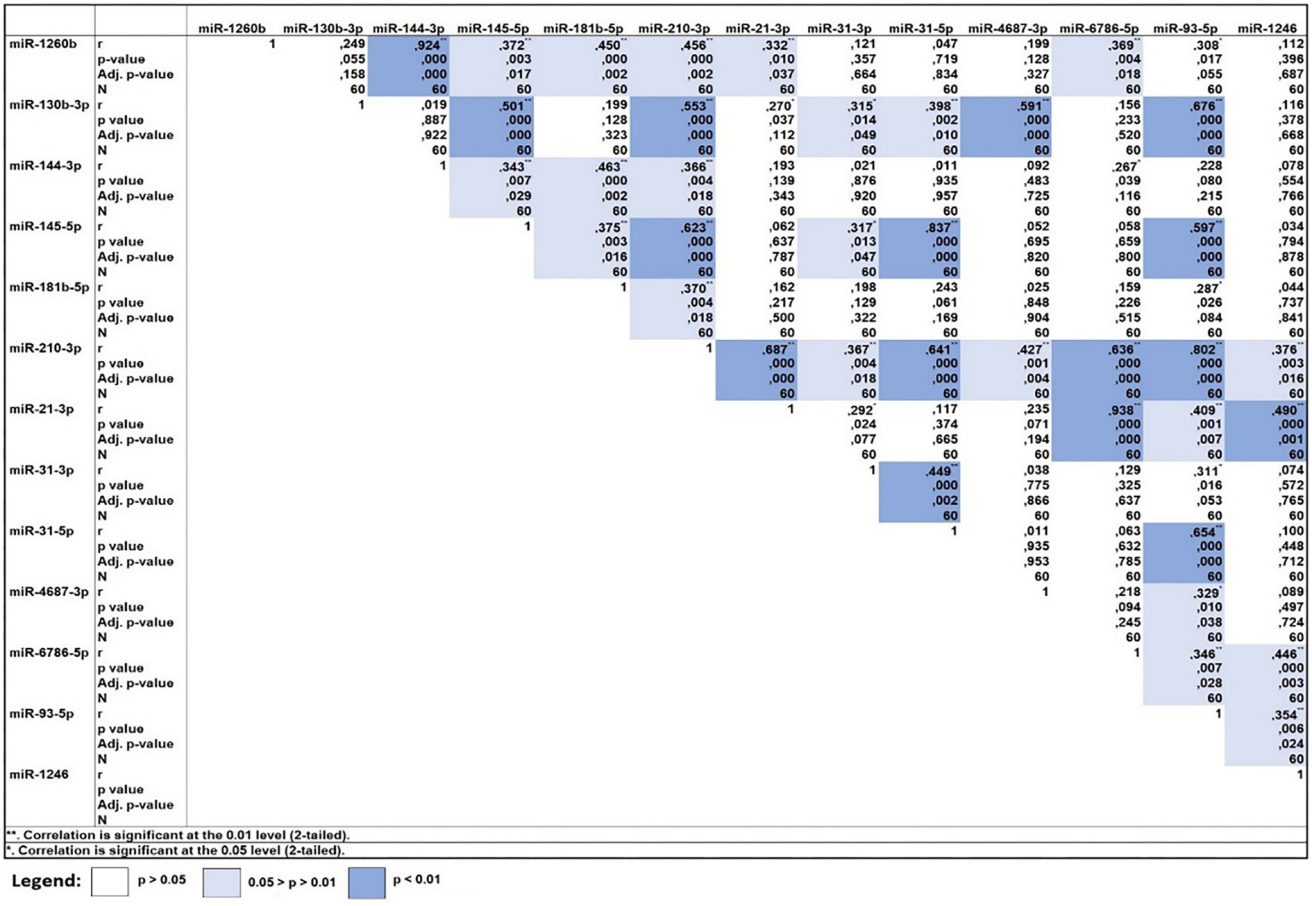
**The correlation matrix of all thirteen significantly deregulated miRNAs in advanced LSCC was generated using RQ values.** Both *P* values and adjusted FDR *P* values were included. Light blue shading and a single asterisk (*) indicate a significance level of < 0.05, while darker blue and double asterisks (**) represent a significance level of < 0.01. White shading indicates non-significance. All correlations were positive, showing moderately strong to robust levels of correlation. Notably, miR-210-3p emerged as a central hub, demonstrating significant associations with all 12 other miRNAs. LSCC: Laryngeal squamous cell carcinoma; miRNAs: MicroRNAs; RQ: Relative quantification; FDR: False discovery rate.

**Figure 2.**
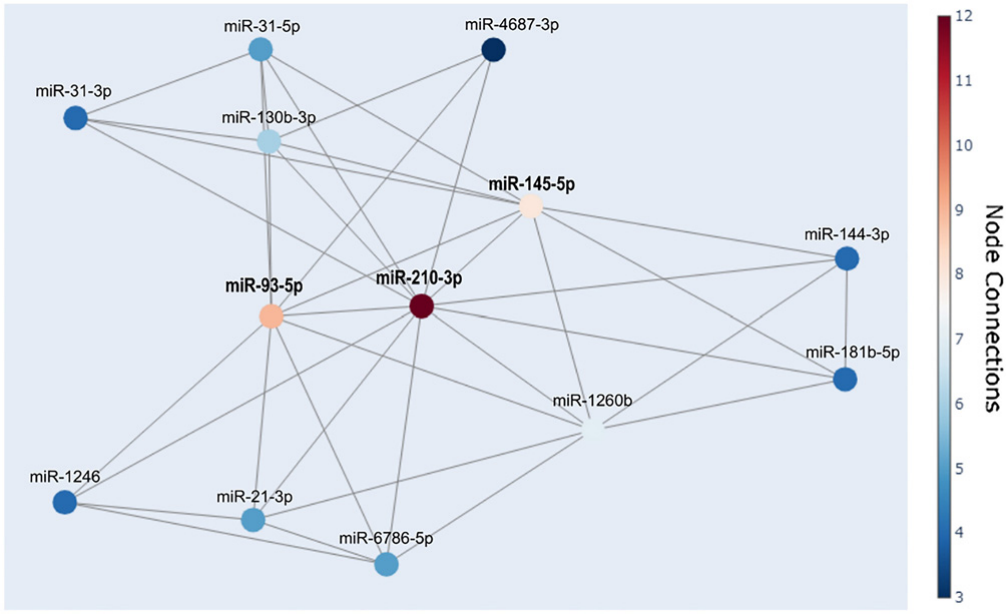
**The correlation network represents significantly associated miRNAs (adjusted FDR *P* values).** The color of the nodes indicates the number of connections. The network highlights miR-93-5p, miR-145-5p, and miR-210-3p, which were analyzed with a greater number of connections: Nine for miR-93-5p, eight for miR-145-5p, and 12 for miR-210-3p. miRNAs: MicroRNAs; FDR: False discovery rate.

### Comprehensive miRNA microarray profiling

In a previous study, we performed miRNA microarray analysis on 12 patients with advanced T4 LSCC using Agilent Technologies’ G3 Human miRNA Microarray Kit (Release 21, 8 × 60 K) [14, 15]. This analysis identified 11 miRNAs (miR-1260b, miR-31-3p, miR-31-5p, miR-93-5p, miR-21-3p, miR-181b-5p, miR-130-3p, miR-145-5p, miR-4687-3p, miR-6786-5p, miR-144-3p, miR-210-3p, and miR-1246) as significantly deregulated. These miRNAs were used to validate findings in a larger cohort of LSCC patients.

### Reverse transcription-quantitative PCR (RT-qPCR)

A total of 500 ng of RNA from each sample was used for complementary DNA synthesis using the miScript II RT Kit (Qiagen, Germany), following the manufacturer’s instructions. Expression analysis of miR-93-5p, miR-145-5p, and miR-210-3p was performed using the miScript SYBR Green PCR Kit (Qiagen, Germany) on a 7900HT Fast Real-Time PCR System (Applied Biosystems, USA). Each reaction was conducted in triplicate, and RNU6-2 (RNA, U6 small nuclear 2) was used as the endogenous control. Table 1 presents the target names, GeneGlobe IDs, and catalog numbers (Qiagen, Germany). Relative quantification (RQ) was calculated using the 2^--ΔΔCt^ method, with RQ values of ≥2 indicating upregulation, values of ≤0.5 indicating downregulation, and values between 1.99 and 0.5 showing no significant alteration.

**Table 2:** Associations between miR-93-5p, miR-145-5p, miR-210-3p and clinicopathological features of all 131 included patients diagnosed with advanced LSCC

**Features**	**N (%)**	**miR-93-5p (RQ)**	**miR-145-5p (RQ)**	**miR-210-3p (RQ)**
Sex*				
Male	127	4.80 ± 8.80	1.01 ± 1.30	3.44 ± 4.40
Female	4	8.19 ± 6.28	0.33 ± 0.15	1.87 ± 0.96
*Age*				
≤ 59	43	4.56 ± 6.69	0.92 ± 0.95	3.69 ± 4.94
> 60	88	6.75 ± 12.45 *P* ═ 0.402	1.07 ± 1.66 *P* ═ 0.283	2.88 ± 3.69 *P* ═ 0.497
*Tumor stage*				
T3	38	4.37 ± 3.03	1.34 ± 1.54	3.42 ± 4.39
T4	93	10.97 ± 2.56 ***P ═ 0.033***	0.77 ± 1.06 *P* ═ 0.053	3.34 ± 4.31 *P* ═ 0.679
*Lymph metastasis*				
N0	77	4.80 ± 8.29	1.16 ± 1.45	1.86 ± 2.25
N1-3	54	5.53 ± 10.61 *P* ═ 0.830	0.56 ± 0.54 *P* ═ 0.095	4.84 ± 6.63 ***P ═ 0.011***
*Differentiation*				
G1	43	5.26 ± 5.97	1.01 ± 1.25	2.99 ± 3.96
G2	73	4.43 ± 8.79	0.79 ± 0.77	4.03 ± 5.03
G3	15	5.79 ± 15.41 *P* ═ 0.889	1.51 ± 2.86 *P* ═ 0.776	2.14 ± 1.69 *P* ═ 0.423
*Sublocalization*				
Supraglottic	57	3.39 ± 5.14	1.07 ± 1.15	3.64 ± 4.55
Transglottic	36	5.99 ± 7.68	0.90 ± 1.50	4.28 ± 5.65
Retrocricoid	20	4.04 ± 9.51	0.33 ± 0.30	1.84 ± 3.52
Subglottic	18	5.77 ± 7.94 *P* ═ 0.819	3.53 ± 6.70 ***P ═ 0.001***	5.88 ± 3.84 ***P ═ 0.002***
Second malignancy*				
Yes	4	1.54 ± 0.52	0.40 ± 0.23	4.40 ± 4.48
No	125	5.19 ± 9.23	2.35 ± 6.05	8.47 ± 2.01
Missing data	2			
*No statistical testing was conducted for the features Sex: Female and Second malignancy: Yes due to the very small size of these patient groups.				

Statistically significant results were reached between miR-93-5p and tumor stage, miR-210-3p and lymph nodal metastasis, and tumor localization with both miR-145-5p and miR-210-3p. LSCC: Laryngeal squamous cell carcinoma.

**Table 3:** The distribution of miRNA expression levels across the patient cohort

**Type of expression**	**miR-93-5p number of patients *n* (%)** *Mean* **±** *SD*	**miR-145-5p** **number of patients *n* (%)**	**miR-210-3p** **number of patients *n* (%)**
Decreased levels	9 (8%) *0.20 ± 0.14*	36 (27%) *0.19 ± 0.13*	19 (14%) *0.22 ± 0.15*
Normal levels	43 (32%) *1.09 ± 0.42*	71 (54%) *0.93 ± 0.38*	48 (37%) *1.10 ± 0.43*
Elevated expression	79 (60%) *10.35 ± 11.43*	24 (19%) *6.15 ± 4.05*	64 (49%) *11.88 ± 14.64*

miRNA: MicroRNA.

### Target gene identification and enrichment analysis

miRNA target genes were predicted using the miRTargetLink 2.0 database for miRNA target prediction [16]. Only strongly predictive and experimentally validated targets were considered for enrichment analysis. Gene enrichment analysis was performed using the GSEApy Python package [17], with interactive visualizations generated using Enrichr [18]. Significantly enriched GO terms and MSigDB Hallmark sets were determined with an adjusted *P* value cut-off of less than 0.05, and a false discovery rate (FDR) of less than 0.05.

### Ethical statement

Informed consent was obtained in writing from all patients, and the documents are stored in the archives of the Molecular Medicine Center and the Department of ENT, Medical University of Sofia. Ethics approval was obtained from the Ethics Committee of the Medical University of Sofia (approval Nos. BK-297/14.04.2015, BK-373/11.04.2016, 7836/17.11.2020, and 4584/04.07.2023).

**Figure 3.**
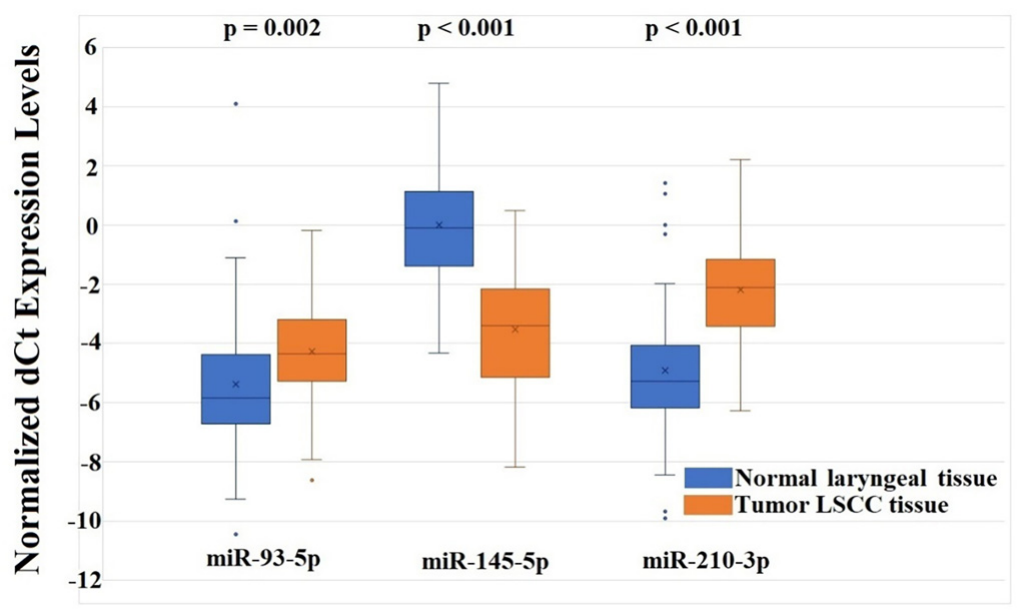
**Normalized dCt expression levels of miR-93-5p, miR-145-5p, and miR-210-3p were analyzed in paired advanced LSCC tumor and normal laryngeal tissues.** The blue boxplots represent normal laryngeal tissue, while the orange boxplots represent tumor tissue. All three miRNAs were significantly deregulated in the *in vivo* tumor samples compared to adjacent normal laryngeal tissues. LSCC: Laryngeal squamous cell carcinoma; miRNAs: MicroRNAs.

**Figure 4.**
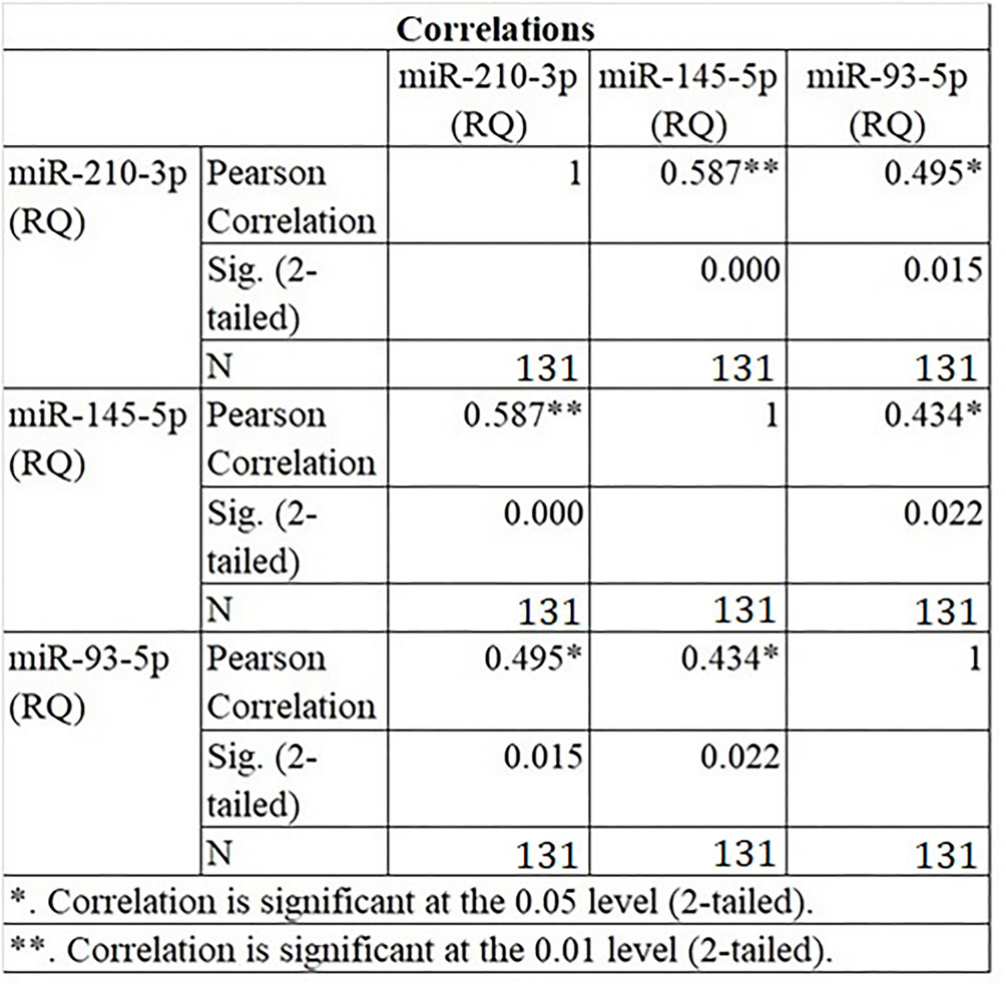
**Pearson’s correlation analysis between miR-93-5p, miR-145-5p, and miR-210-3p in the validation group of advanced LSCC patients (*n* ═ 131).** All three miRNAs showed positive and significant moderately strong correlations with linear relationships. LSCC: Laryngeal squamous cell carcinoma; miRNAs: MicroRNAs.

### Statistical analysis

SPSS v27 software (IBM, USA) was used for statistical analysis. Data normality was evaluated using the Kolmogorov–Smirnov test. Statistical analyses included *t*-tests, one-way ANOVA, Pearson correlation, and Benjamini–Hochberg FDR correction where appropriate [19]. Statistical significance was defined as an adjusted *P* value of less than 0.05. Correlation networks were visualized using Plotly [20], with scatterplots, regression lines, and slopes depicting the strongest correlations.

## Results

### Co-expression analysis of miRNAs

The main objective of this study was to analyze the most co-expressed miRNAs from the previously identified significantly deregulated miRNAs in an advanced LSCC group [14, 15]. To examine the strength and direction of relationships between miRNA variables, we employed Pearson’s correlation analysis. Despite known limitations, such as its assumption of normal distribution and sensitivity to outliers, Pearson’s method offers advantages in high-throughput datasets, particularly when sample sizes exceed 30, per the central limit theorem. We also considered Pearson’s correlation as a precursor to constructing a co-expression network [21, 22]. In this analysis, all 13 miRNAs previously validated as significantly deregulated in advanced LSCC were included [15]. The Benjamini–Hochberg FDR correction was applied to account for multiple comparisons. Remarkably, all correlations were positive, with several exhibiting moderate to strong relationships (Figure 1). This correlation data was used to construct a correlation network (Figure 2), where miR-210-3p emerged as a central hub, demonstrating positive correlations with all 12 other miRNAs. Notably, strong co-correlations were observed between miR-93-5p, miR-145-5p, and miR-210-3p, each with significant node connections—nine, eight, and 12, respectively. These three miRNAs were identified as key co-expressed miRNAs and were subsequently validated in an *in vivo* advanced LSCC sample group. We also performed bioinformatics and computational analyses to uncover their biological relationships in advanced LSCC.

**Figure 5.**
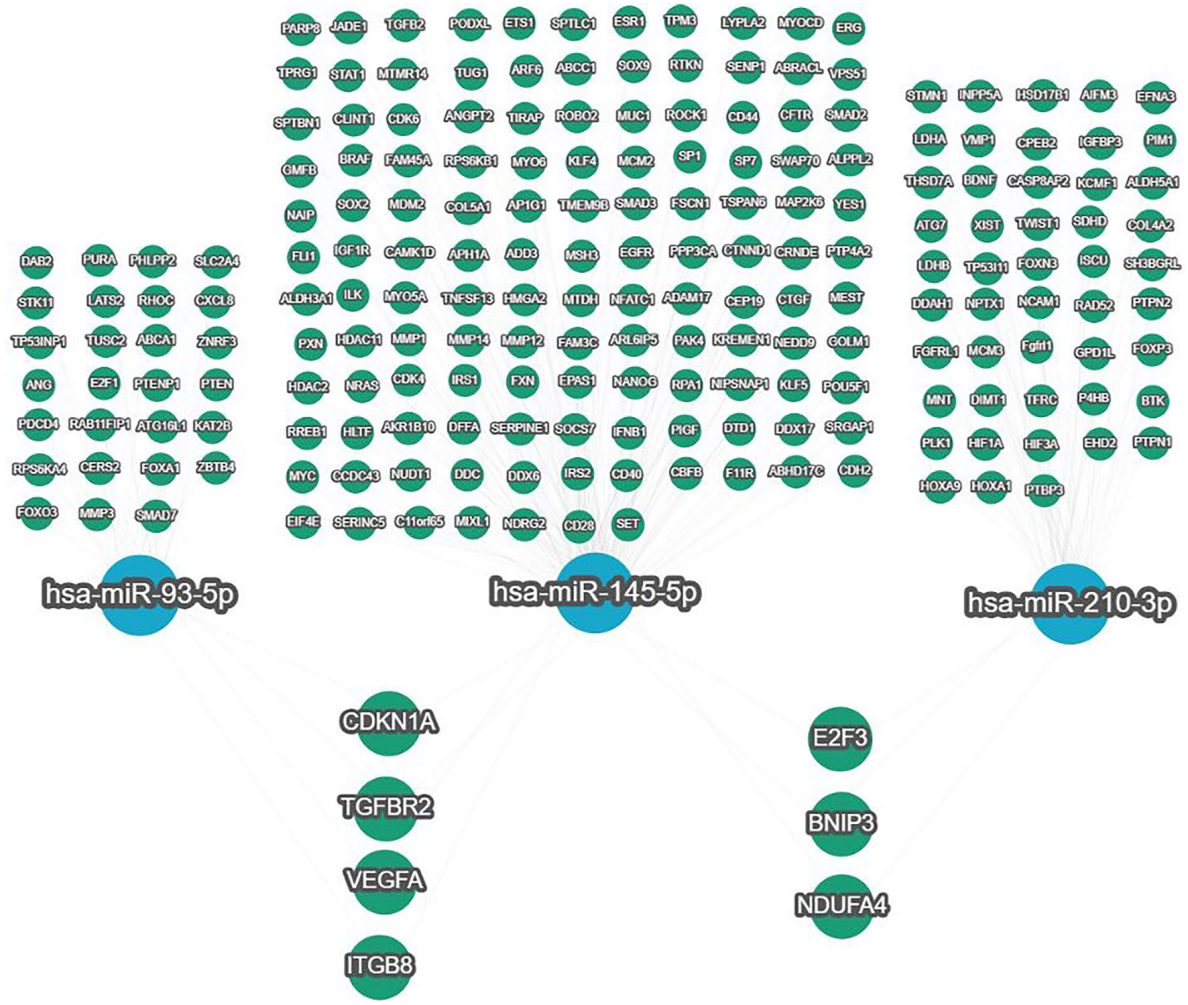
**Strong, experimentally validated targets of miR-93-5p, miR-145-5p, and miR-210-3p were identified using the miRTargetLink 2.0 web-based tool.** Common targets between miR-93-5p and miR-145-5p included *CDKN1A*, *TGFBR2*, *VEGFA*, and *ITGB8*. Common targets between miR-145-5p and miR-210-3p were *E2F3*, *BNIP3*, and *NDUFA4*. No common targets were found between miR-93-5p and miR-210-3p.

### *In vivo* relative expression and correlation analysis of miR-93-5p, miR-145-5p, and miR-210-3p in validation advanced LSCC group samples

The analyzed co-expressed set of miR-93-5p, miR-145-5p, and miR-210-3p was explored in a validation group consisting of 131 patients diagnosed with advanced LSCC. The majority of patients (*n* ═ 93) were classified as T4 stage, while 38 were classified as T3. Positive lymph node metastasis was detected in 54 patients, while 77 were lymph node metastasis-negative. Forty-three samples were well-differentiated carcinoma (G1), 73 were moderately differentiated (G2), and 15 were poorly differentiated (G3). The tumor sites included: transglottic (36), supraglottic (57), subglottic (18), and retrocricoid carcinoma (20). In four LSCC patients, a secondary malignancy (lung cancer) was identified. The mean age of the validation group was 64.62 ± 8.68 (range: 46–83 years), consisting of four females and 127 males. All clinicopathological features are listed in Table 2. Expression testing and statistical analysis revealed that all three co-expressed miRNAs were significantly deregulated in advanced laryngeal tumor samples compared to adjacent healthy tissue. miR-93-5p was generally overexpressed, with mean RQ levels of 6.04 (min: 0.04, max: 46.13) in the validation group. miR-145-5p was underexpressed, with mean RQ levels of 1.55 ± 4.10 (min: 0.18, max: 19.936). miR-210-3p was overexpressed, with mean RQ levels of 3.97 ± 5.96 (min: 0.006, max: 17.789). A detailed breakdown of miRNA expression levels across patients is shown in Table 3. Reversed and normalized dCt values were used for visualization, and the results are presented as boxplots (Figure 3), including *P* values. Pearson’s correlation analysis indicated that all three miRNAs were positively and significantly associated with moderately strong linear relationships in the validation group (*n* ═ 131). miR-93-5p and miR-145-5p had a correlation coefficient of *r* ═ 0.434 (*P* ═ 0.022), miR-93-5p and miR-210-3p showed *r* ═ 0.495 (*P* ═ 0.015), and the highest correlation was between miR-145-5p and miR-210-3p with *r* ═ 0.587 (*P* < 0.001) (Figure 4). While the correlation strengths in the validation group were slightly lower than the initial data, they still indicated moderately strong relationships, fully consistent with prior results (Figure 1). The correlation analysis reinforced the potential synergism in miRNA expression and their critical role in advanced LSCC carcinogenesis.

**Figure 6.**
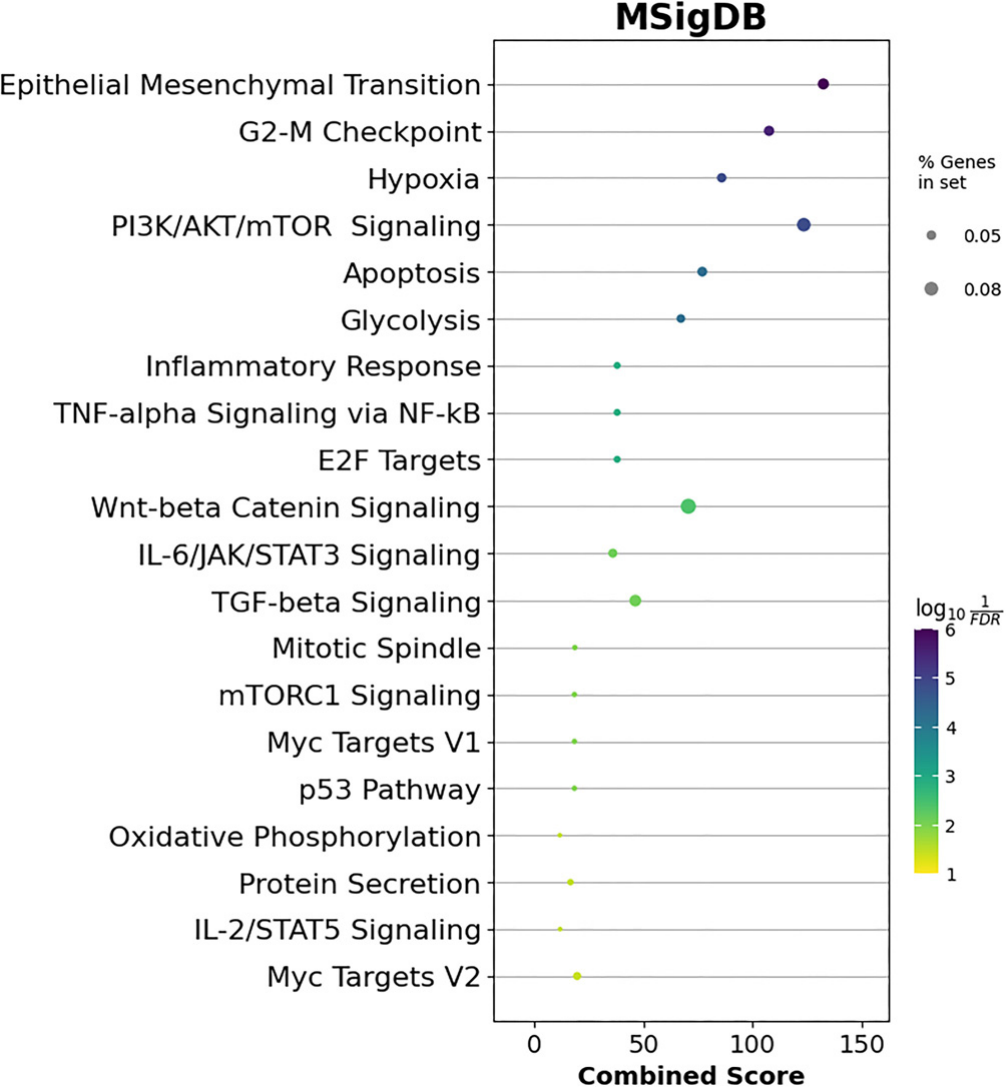
**MSigDB pathway enrichment analyses were performed on the differentially expressed strong target genes of the miR-93-5p/miR-145-5p/miR-210-3p set.** The top 30 significantly deregulated pathways were included in the figure. The *P* values were visualized as log 10 (adjusted FDR *P* values), with colors ranging from dark blue to yellow, where dark blue indicates greater significance. The MSigDB results were statistically analyzed using The Human MSigDB v2023.1 via the GSEApy Python package. The “% Genes in set” represents the percentage of target genes that overlapped with the pathway of interest. The *x*-axis shows the combined score as defined by Enrichr [18]. FDR: False discovery rate.

### Identification of common RNA targets of miR-93-5p/miR-145-5p/miR-210-3p set

To identify shared RNA targets of miR-93-5p, miR-145-5p, and miR-210-3p, we used the miRTargetLink 2.0 web-based tool. Our analysis focused on experimentally validated, strongly predictive targets. In total, 209 strong targets were analyzed for the three miRNAs (Supplementary File  1: Strong targets). Common targets were found between miR-93-5p and miR-145-5p (Supplementary File 2: Common strong targets), including *CDKN1A*, *TGFBR2*, *VEGFA*, and *ITGB8*. miR-145-5p and miR-210-3p shared common targets, such as *E2F3*, *BNIP3*, and *NDUFA4* (Figure 5). However, no common targets were found between miR-93-5p and miR-210-3p or across all three miRNAs. These strong targets were subjected to further functional enrichment analysis.

### Functional enrichment of key miRNA strong target genes

Using MSigDB pathway enrichment analysis, we identified 20 significantly enriched pathways based on adjusted FDR *P* values (Figure 6). These pathways included epithelial–mesenchymal transition (EMT), the G2-M checkpoint, hypoxia, PIK3/AKT/mTOR signaling, apoptosis, and others. Key genes associated with these pathways included *MMP* family members, *HIF1A*, *VEGFA*, *PLK1, MYC, E2Fs, EGFR, TGFB2*, and the *SMAD* family. A full list of enriched MSigDB pathways is available in Supplementary File 3.

**Figure 7.**
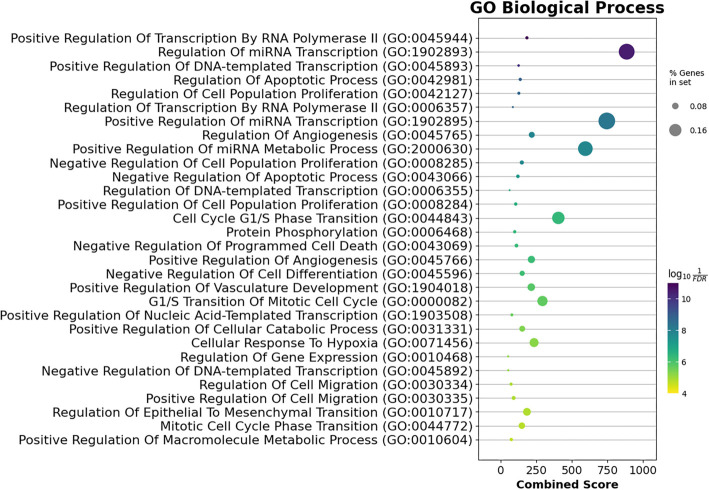
**The BP over-representation analysis of the strong target genes of the miR-93-5p/miR-145-5p/miR-210-3p set shows the top 15 significantly enriched GO BP terms.** “% Genes in set” represents the percentage of target genes that overlapped with the term of interest. The *x*-axis displays the combined score as defined by Enrichr [18]. BP: Biological process; GO: Gene Ontology.

**Figure 8.**
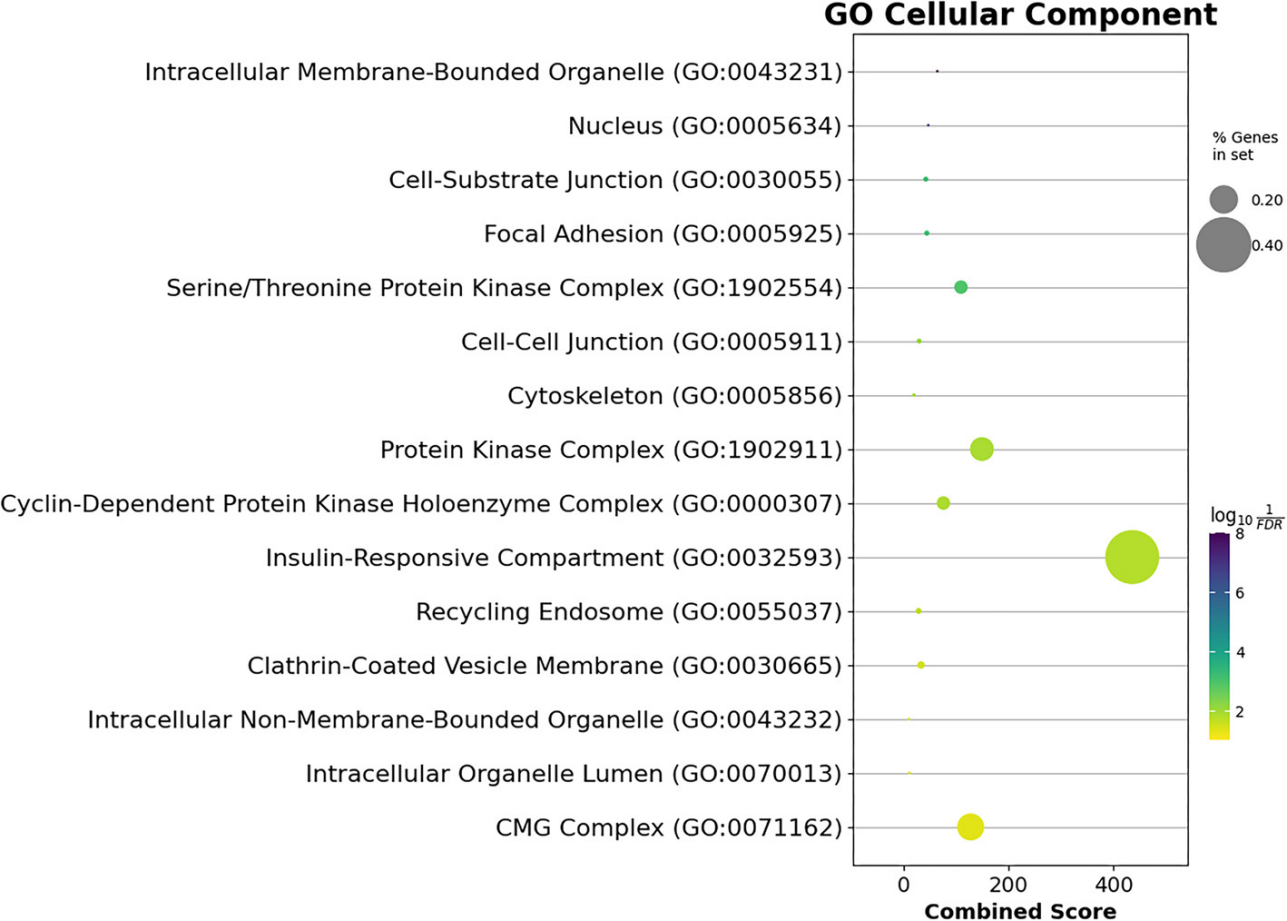
**The CC over-representation analysis of the strong target genes of the miR-93-5p/miR-145-5p/miR-210-3p set shows the top 30 significantly enriched GO CC terms.** “% Genes in set” represents the percentage of target genes that overlapped with the term of interest. The *x*-axis displays the combined score as defined by Enrichr [18]. GO: Gene Ontology; CC: Cellular component.

**Figure 9.**
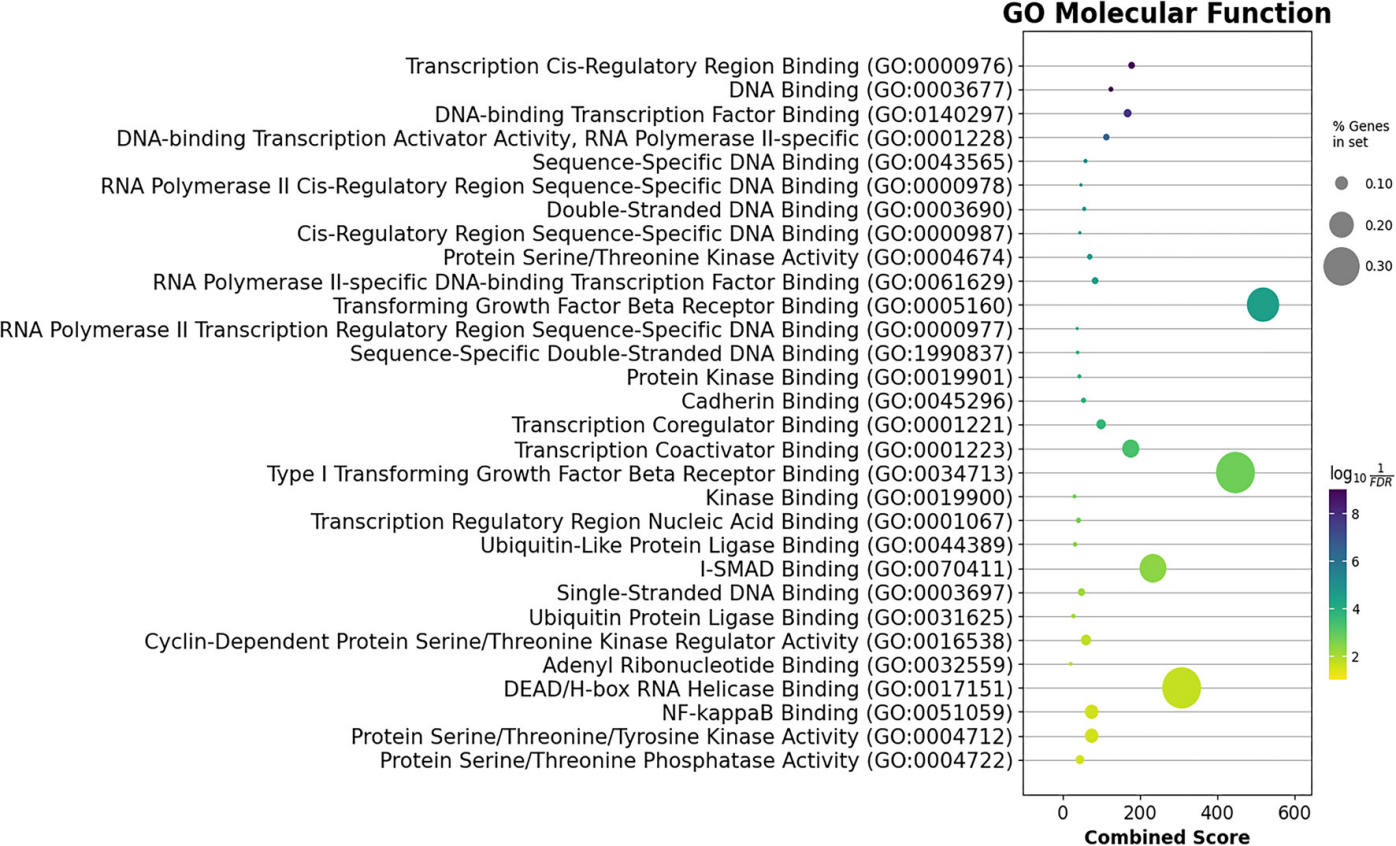
**The MF over-representation analysis of the strong target genes of the miR-93-5p/miR-145-5p/miR-210-3p set shows the top 30 significantly enriched GO MF terms.** “% Genes in set” represents the percentage of target genes that overlapped with the term of interest. The *x*-axis displays the combined score as defined by Enrichr [18]. MF: Molecular function; GO: Gene Ontology.

GO analysis also identified top enriched GO terms across BP, molecular functions (MFs), and cellular component (CC) categories. A total of 429 BP terms were analyzed (Supplementary File 4), with the top 30 presented in Figure 7. These terms included transcription regulation, apoptosis, angiogenesis regulation, cell cycle G1/S phase transition, and hypoxia response. In the CC category (Supplementary File 5), 15 significant terms were enriched, including intracellular membrane-bounded organelles, the nucleus, cell–cell junctions, and protein kinase complexes (Figure 8). In the MF category, 45 significantly enriched terms were identified (Supplementary File 6), with the top 30 terms highlighted in Figure 9. These included DNA binding, cadherin binding, protein kinase binding, ubiquitin-like protein ligase binding, and ATP binding. Notable targets affected by these enriched terms included the *FOXO* family, *EPAS1*, *TWIST1*, *ETS1*, *EGFR*, the *SOX* family, the *SMAD* family, and the *E2F* family.

### Association between miR-93-5p, miR-145-5p, miR-210-3p expression levels and clinicopathological features

The association between the RQ data for the three miRNAs and the clinicopathological features of the LSCC patients is summarized in Table 2. The study included 131 patients diagnosed with advanced LSCC. The clinicopathological features investigated were sex, age, tumor stage, lymph node metastasis, cell differentiation, tumor location, and the presence of a second malignancy. All patients were positive for tobacco use and alcohol consumption, which strongly correlates with advanced LSCC. Our data showed a statistically significant association between miR-93-5p expression levels and tumor stage, with higher miR-93-5p levels observed in T4 compared to T3 patients (*P* ═ 0.033). miR-210-3p expression was significantly higher in patients with lymph node metastasis (N1-3) compared to those without (N0) (*P* ═ 0.011). Both miR-145-5p and miR-210-3p showed significant associations with tumor location (*P* ═ 0.001 and *P* ═ 0.002, respectively). Other clinicopathological features did not show statistically significant associations. Due to extreme variance between subgroups, the associations between sex and second malignancy were excluded from statistical analysis.

## Discussion

This study is the first to deeply investigate the role of three miRNAs—miR-93-5p, miR-145-5p, and miR-210-3p—in a large cohort of *in vivo* samples from 131 patients with advanced LSCC. We evaluated their co-expression levels, correlation potential, and the known targets of significantly deregulated cellular pathways using in silico methods. Recent studies have highlighted the benefits of examining multiple miRNAs in carcinogenesis rather than focusing on a single miRNA. This is because miRNA expression levels can fluctuate significantly depending on the physiological environment. A critical aspect of understanding miRNA function lies in identifying their target mRNAs and the pathways they regulate [23]. We incorporated the relative expression levels of all 13 miRNAs (miR-1260b, miR-31-3p, miR-31-5p, miR-93-5p, miR-21-3p, miR-181b-5p, miR-130-3p, miR-145-5p, miR-4687-3p, miR-6786-5p, miR-144-3p, miR-210-3p, and miR-1246), previously identified as significantly deregulated in our earlier studies on advanced LSCC samples [14, 15]. Our correlation analysis centered on miR-93-5p, miR-145-5p, and miR-210-3p, with miR-210-3p emerging as a central hub within the correlation network. These findings underscore the potential importance of these three miRNAs in LSCC. Using the miRTargetLink 2.0 database, we identified 209 strong targets for the miR-93-5p/miR-145-5p/miR-210-3p set, which were then subjected to enrichment analysis. Pathway enrichment analysis using the MSigDB database, along with GO enrichment analysis**.** Notably, we filtered enrichment data based on adjusted FDR *P* values, removing pathways unrelated to LSCC or head and neck cancer. Our results highlighted that key deregulated processes included cell cycle regulation (G2-M checkpoint, mitotic spindle, *E2F* targets), hypoxia, apoptosis, cell metabolism (glycolysis, oxidative phosphorylation, protein secretion), cell proliferation (PI3K/AKT/mTOR signaling, mTORC1 signaling, TGF-beta signaling), and invasion/metastasis pathways (TNF-alpha signaling via NF-kB, IL-6/JAK/STAT3 signaling). Additionally, the p53 pathway was implicated. These processes are all closely linked to cell cycle regulation, DNA repair, apoptosis, autophagy, and metabolism. Our findings suggest that these three miRNAs play critical roles in cancer progression, tumor staging, and nodal metastasis. They may also influence tumor location, as significant associations with clinicopathological features were identified (Table 2). In our bioinformatic analysis, we found that miR-145-5p regulates *MDM2*, while miR-210-3p targets *TP53I11*, a gene induced by p53 that is predicted to be involved in p53-mediated apoptosis [24]. A notable co-expression exists between *TP53* and *MDM2*, a complex associated with tumor aggressiveness [25]. Additionally, our correlation analysis showed a significant linear correlation between miR-145-5p and miR-210-3p, further supporting their involvement in the dynamics of laryngeal tumorigenesis. The PI3K/Akt signaling pathway is of particular interest due to its role in LSCC progression, proliferation, and metastasis [26], as well as its involvement in *VEGFR2*-mediated angiogenesis [27]. miR-93 activates this pathway by downregulating tumor suppressor genes, such as *LKB1*, *PTEN*, and *CDKN1A*, thereby stimulating PI3K/Akt signaling [28]. miR-145 has also been shown to influence PI3K/Akt signaling in both LSCC [29] and esophageal SCC (ESCC) [30]. Downregulation of miR-145 leads to enhanced tumor growth, invasion, metastasis, and cancer stem cell (CSC) proliferation, while inhibition of PI3K/Akt signaling has been associated with reduced multidrug resistance and improved responses to cisplatin treatment [31]. The TGF-beta, Wnt-beta-catenin, and IL-6/JAK/STAT3 signaling pathways are involved in metastasis (Figure 4). miR-93 regulates the TGF-beta pathway by downregulating *NEDD4L*, an E3 ubiquitin ligase that stimulates EMT [32]. miR-93 also affects the Wnt pathway, a critical pathway in many cancers, by downregulating *ZNRF3*, an inhibitor of Wnt signaling [33]. Hypoxia, a common feature of solid tumors, including LSCC, is associated with radiotherapy resistance and poor prognosis in head and neck cancers [34]. *HIF-1α*, a transcription factor upregulated under hypoxia, is overexpressed in advanced LSCC, contributing to tumor aggressiveness [35]. *HIF-1α* not only promotes tumor cell proliferation and invasiveness but also enhances immune evasion by upregulating *PD-L1* [36]. The regulation of miR-145 and miR-210 by HIF-1α suggests their roles in therapy resistance, including resistance to radiotherapy and chemotherapy. miR-93 also plays a role in the HIFα switch, which occurs in prolonged hypoxic conditions [37]. Several studies have linked miR-210 to resistance to radiotherapy and chemotherapy by targeting genes involved in radioresistance, DNA repair, and chemoresistance [38–40]. Additionally, miR-93-5p has been implicated in the regulation of carcinogenesis and tumor immunity by targeting the *PD-L1/CCND1* axis, with potential use as a biomarker for patient risk stratification and treatment [41]. miR-145-5p may enhance the effectiveness of the only FDA-approved targeted anti-EGFR therapy, cetuximab, by increasing antibody-dependent cellular cytotoxicity [42].

Further research is needed to validate these findings and explore the possibility of using these miRNAs as biomarkers for disease prognosis and treatment response. This study opens new avenues for personalized medicine in the management of advanced LSCC, with the potential for improved patient outcomes and treatment strategies. Our next goal is to apply the findings from this study in future *in vitro* and *ex vivo* experiments to identify miRNAs with the greatest potential as biomarkers for advanced LSCC.

## Conclusion

Our results highlight the significant roles of miR-93-5p, miR-145-5p, and miR-210-3p in regulating pathways related to the cell cycle, hypoxia, metabolism, apoptosis, angiogenesis, and metastasis. These miRNAs are closely associated with tumor grade, nodal metastasis, and tumor localization, indicating their involvement in advanced laryngeal carcinogenesis. Furthermore, they affect the efficacy of existing chemotherapies and may contribute to radiotherapy resistance. miR-145-5p, in particular, influences responses to anti-EGFR and PD-1 immunotherapies, while all three miRNAs impact CSC proliferation pathways, potentially driving the progression of advanced LSCC. Investigating these key regulatory miRNAs is crucial for the future management of LSCC, as a small subset of cancer cells may survive treatment, contributing to resistance and disease progression.

## Supplemental data

All analyzed strong targets of miRNA-93-5p/miR-145-5p/miR-210-3p set and pathway and GO terms are provided in supplementary excel file at the following link: https://www.bjbms.org/ojs/index.php/bjbms/article/view/10947/3505

## Data Availability

Full data are available on request.
